# Analysis of clinical parameters as predictors of hearing recovery in patients with sudden sensorineural hearing loss

**DOI:** 10.1016/j.bjorl.2022.08.002

**Published:** 2022-08-19

**Authors:** Nikola Kolja Poljak, Marisa Klančnik, Petar Ivanišević, Petra Mikulić, Marta Zrinka Vucemilovic

**Affiliations:** a University Hospital of Split, Department of Otorhinolaryngology-Head and Neck Surgery, Split, Croatia; b Sunset Medical Care, General Practice, Florida, USA

**Keywords:** Sudden sensorineural hearing loss, Predictors, Hearing recovery, Diabetes mellitus, Tone audiometry

## Abstract

**Objective:**

To investigate the impact of certain clinical parameters on likelihood of hearing recovery after SSNHL and to detect potential variables that predict its outcome.

**Methods:**

A retrospective study was conducted in order to identify the clinical parameters which influence hearing recovery after SSNHL. Medical charts of 87 patients diagnosed with SSNHL and treated in the clinic from January 2015 to December 2019 were retrospectively reviewed. The effects of several parameters on the success of the treatment were statistically evaluated. Such parameters studied were age, gender, the severity of hearing loss, audiometric curve pattern, the time treatment was initiated, and metabolic factors.

**Results:**

Patient categorization according to tonal audiometric curve results statistically differed in terms of recovery (complete, partial, no recovery) (χ^2^ = 32.5; *p* <  0.001). There are 2× more diabetic patients in the group no recovery than in the group partial recovery (OR = 3.1; 95% CI 0.95‒10; *p* =  0.061), and 5.3× more than in the group complete recovery (OR = 10.4; 95% CI 2.3‒45; *p* =  0.002) (χ^2^ = 11.2; *p* =  0.004). There is a statistically significant correlation between onset of therapy and recovery on a scale of significance of 93% (χ^2^ = 5.3, *p* =  0.069).

**Conclusion:**

In our study tonal audiometry results, diabetes mellitus and onset of therapy were shown to be a statistically significant negative predictive factors for recovery. Results of pure tone audiometry suggest a flat profound curve is statistically associated with the no recovery rates, and a down sloping curve proved to be statistically associated with complete recovery rates.

**Level of evidence:**

Level 1.

## Highlights

• Sudden sensorineural hearing loss is defined as a sensoneural hearing loss of at least 30 decibels, which affects at least three consecutive frequencies within 72 hours.
• The etiology of SSNHL is fairly unknown, and so, it is often referred to as an idiopathic disease.
• Sudden sensorineural hearing loss requires immediate treatment.
• Tonal audiometry results, diabetes mellitus and onset of therapy were shown to be a statistically significant negative predictive factors for recovery.

## Introduction

Sudden Sensorineural Hearing Loss (SSNHL), first described in 1944 by De Klein, is defined as a sensoneural hearing loss of at least 30 decibels (dB), which affects at least three consecutive frequencies within 72 hours. It is a common otological emergency, which incidence rate ranges between 5 and 20 cases for every 100,000 individuals per year, and it requires immediate treatment. The majority of cases of SSNHL are unilateral, with bilateral hearing loss occurring only in approximately 1%‒2% of cases.[1] The hearing impairment experienced in this disease can also be accompanied by tinnitus, vertigo, and a sensation of aural fullness.[2] The etiology of SSNHL is fairly unknown, and so, it is often referred to as an idiopathic disease. In fact, less than one third of all cases can be definitely attributed to viral infection, vascular impairment, autoimmunity, or endocochlear membrane rupture.[3]

In addition to its frequently uncertain origins, the recovery from SSNHL is, too, often uncertain. Spontaneous recovery of the hearing threshold can be observed, however, only in about 35% to 65% of cases. The real number of patients with spontaneous recovery from SSNHL is currently unknown, due to the fact that many who recovered spontaneously within the first few days did not seek medical treatment, and therefore, were not considered into the statistic. With this unavailability of true numbers, there remains a controversy of statistics between cases of spontaneous recovery and the efficacy of early medical therapy.[4], [5], [6] Despite these controversies in the treatment of SSNHL, systemic corticosteroid treatment is still the most accepted therapy. Ultimately, outcome of SSHL, especially of idiopathic origin, is unpredictable.[7], [8]

Numerous studies have attempted to establish a relationship between certain clinical parameters and the degree of hearing improvement, each often resulting with different conclusions. Several prognostic factors have also been investigated in various studies, including gender and age of the patient, presence of concomitant vestibular symptoms and tinnitus, degree of hearing loss, audiometric configuration, the time of initiation of therapy, and metabolic factors. However, there remains a lack of consensus regarding the influence of these factors on the clinical outcome.[9], [10], [11]

Therefore, it is the purpose of this retrospective study to further investigate the impact of certain clinical parameters on likelihood of hearing recovery after SSNHL, and to detect potential variables that predict its outcome.

## Methods

A retrospective study was conducted in order to identify the clinical parameters which influence hearing recovery after SSNHL. Medical charts of 87 patients diagnosed with SSNHL in the clinic from January 2015 to December 2019 were retrospectively reviewed. All patients were both diagnosed and treated at the Department of Otorhinolaryngology and Head and Neck Surgery. This study was approved by the institution`s Ethics Committee.

The effects of several parameters on the success of the treatment were statistically evaluated. Such parameters studied were age, gender, the severity of hearing loss, audiometric curve pattern, the time treatment was initiated, and metabolic factors.

Patients with bilateral sudden hearing loss, hearing loss that had begun more than 30 days prior to their examination, those younger than 15 years old, Meniere`s disease, ear trauma, patients affected by ototoxicity and neoplasms were excluded from the study.

Patients were evaluated using method of pure tone threshold audiometry. Pure Tone Average (PTA) was calculated as the mean of thresholds at six frequencies (250, 500, 1000, 2000, 4000 and 8000 Hz).

For treatment, all patients received the same intravenous methylprednisolone treatment beginning with 250 mg, tapering every two days for 10 days. Those with diabetes underwent additional frequent blood glucose monitoring, and, according to the glucose values, insulin was given.

Based on pure tone audiometry of the pre-treatment audiometric test, patients were divided into four audiometric curve groups: up-sloping (low frequencies affected), down-sloping (high frequencies affected), flat moderate to severe (all frequencies involved with PTA between 40 and 90 dB) and profound (flat audiogram with PTA more than 90 dB).

Hearing recovery was evaluated by measuring the hearing level at the first day and after three months. Hearing improvement was assessed by modifying Siegel’s criteria.[12]

The outcomes were classified as complete recovery (PTA < 25 dB or identical to the contralateral, non affected ear), partial recovery consists of marked improvement (PTA improvement > 30 dB) and slight improvement (PTA improvement between 19 and 30 dB), and no recovery (PTA improvement < 10 dB).

Statistical analysis was done by use of IBM SPSS version 20.0 (IBM Corp. Armonk, NY, USA).

Statistical siginficance was set to *p* < 0.05 and all confidence intervals were given at the 95%. A Scapiro-Wilk test indicated statistical significant deviations from normal distribution of all numeric variables, the median and interquartile range were used.

Analysis of statistical significance of differences in numeric variables between complete and incomplete study groups was performed by Mann-Whitney *U*-test.

Statistic significance of the differences of categorical demographic and clinical characteristics was calculated by the Chi-Square (χ^2^) test. We also use univariate and multivariate binary logistic regression.

## Results

This study included a sample of 84 patients from the Otorhinolaryngology department, who between 2015 and 2019, were diagnosed with and treated for sudden sensorineural hearing loss. The sample included 50 (60%) males and 34 (40%) females. The median age of both the male and female patients was 56 years (Q1‒Q3: 45‒66; min-max: 8‒87); (Males: Q1‒Q3: 45‒62); (Females: Q1‒Q3: 47‒70 years; min‒max: 27‒84). Therefore, the males and females did not statistically differ by age (Mann-Whitney test: z = 0.862; *p* = 0.389).

In Table 1, the clinical and demographic parameters of the sampled patients are displayed.

**Table 1:** Clinical and demographic data of the sampled patients with sudden sensorineural hearing loss (n = 94).

Clinical and demographic data	
Gender; n (%)	
Males	50 (60)
Females	46 (40)
Age (years) median (Q1‒Q3); min‒max	56 (45‒66; 8‒87)
Dyslipidemia; n (%)	52 (62)
Hypertension; n (%)	44 (52)
Diabetes; n (%)	22 (26)
Onset of therapy (days) median (Q1‒Q3; min‒max)	3 (1‒6;1‒21)
Curve shape; n (%)	
Flat profound	25 (29.8)
Down sloping	32 (38.1)
Up sloping	21 (25)
Flat moderate to severe	6 (7.1)
Hearing loss recovery; n (%)	
Complete recovery	31 (37)
Partial recovery	34 (40)
No recovery	19 (23)

Of the total sampled patients (n = 84), 31 (37%) completely recovered. Of the remaining 53 (63%), 19 patients did not recover, while 34 experienced incomplete recovery.

In Table 2, associative risk factors are displayed in comparison to the extent of disease recovery.

**Table 2:** Clinical and demographic characteristics of patient patients with sudden sensorineural hearing loss according to recovery (complete or incomplete).

	Hearing loss recovery					
	Total	Complete (n = 31)	Incomplete (53)	*p*^a^	OR (95% CI)	*p*^b^
Gender; n (%)				0.801		
Males	50 (60)	19 (61)	31(58)			
Females	46 (40)	12 (39)	22 (41)			
Age (years)				0.175		
≤ 56	42 (50)	19(61)	23 (43)			
> 56	42 (50)	12 (39)	30 (57)			
Dyslipidemia; n (%)	52 (62)	17 (55)	35 (66)	0.431		
Hyperthension; n (%)	44 (52)	17 (55)	27 (51)	0.906		
Diabetes; n (%)	22 (26)	3 (10)	19 (36)	0.018	5.2(1.4‒19)	0.014
Onset of therapy (days)				0.045	3.1 (1.1‒8.3)	0.028
1‒3	52 (62)	24 (77)	28 (53)			
> 3	32 (38)	7 (23)	25 (47)			
Curve shape; n (%)				<0.001		
Flat profound	25 (29.8)	3 (9.7)	22 (41.5)			
Down sloping	32 (38.1)	14 (45.2)	18 (34)			
Up sloping	21 (25)	14 (45.2)	7 (13.2)			
Flat moderate to severe	6 (7.1)	0	6 (11.3)			
Curve shape; n (%)				<0.001	10.4 (2.8‒3.6)	<0.001
Down sloping and up sloping	53 (63)	28 (90)	25 (47)			
Flat profound and flat moderate to severe	31 (37)	3 (10)	28 (53)			

Note: Age is categorized into two groups according to median age ≤ 56 i > 56.

Onset of therapy is divided into two groups according to the median days ≤ 3 i > 3.

a χ^2^ test.

b Logistic regression.

According to the data, neither gender (χ^2^ = 0.064; *p* = 0.801), age 65; > 65(χ^2^ = 1.8; *p* = 0.175), dyslipidemia (χ^2^ = 0.620, *p* =  0.431), nor hypertension (χ^2^ = 0.014, *p* =  0.906) was a statistically significant predictive factor for specific recovery expectations.

There is a 3.6× greater number of diabetic patients found in the group incomplete recovery than in the group of complete recovery (χ^2^ = 5.6; *p* =  0.018).

The ratio of incomplete and complete recovery was 5× higher in patients with diabetes then in patients without diabetes (OR = 5.2; 95% CI 1.4‒19; *p* =  0.014).

The sampled patients whose therapy began after 3 days have 2× more cases of incomplete recovery than complete recovery (χ^2^ = 2.4, *p* =  0.045).

The ratio of incomplete and complete recovery was 3.1× higher in patients began therapy after three days then in patients who began within 3 days (OR = 3.1; 95% CI 1.1–8.3, *p* =  0.028).

Patient categorization according to tonal audiometric test results is a statistically significant predictive factor for hearing loss recovery (χ^2^ = 18.8; *p* < 0.001). Not a single patient with a type flat moderate to severe curve experienced complete recovery. Patients with a type flat moderate to severe curve had 4.3× more cases of incomplete recovery than complete recovery. Those patients with a type of down sloping curve had 3.4× more cases of complete recovery than incomplete recovery.

Upward and down sloping curves were not statistically comparable in terms of recovery (χ^2^ = 1.83; *p* = 0.176). In further analysis, we will monitor them together in association with recovery. Curves type flat profound and flat moderate to severe have shown to be negative predictive factors for recovery, and so in further analysis, they will be considered as one group.

There is a statistically significant difference between tonal audiometric curve results (down sloping and up sloping; flat profound and flat moderate to severe) and recovery (χ^2^ = 13.8, *p* < 0.001). Patients with down sloping and up sloping curves experienced 1.9× more cases of complete recovery than incomplete recovery. In comparison, patients with flat profound and flat moderate to severe curve resulted in 5.3× more cases of incomplete recovery than complete recovery.

The ratio of incomplete and complete recovery was 10× higher in patients with curves type flat profound and flat moderate to severe then in patients with curve types down sloping and up sloping (OR = 10.4; 95% CI 2.8‒3.6; *p* < 0.001).

Variables which the univariate logistic regression analysis displayed as statistically significant factors for recovery were included into the multivariate logistic regression, in which the dependent variable is recovery (complete or incomplete). These variables included were Diabetes Mellitus, tonal audiometric curve results (down sloping and up sloping; flat profound and flat moderate to severe), and onset of therapy (≤ 3, > 3 days) (Table 3).

**Table 3:** Multivariate logistic regression for incomplete recovery in regard to complete recovery.

	OR	95%CI	*p*
Diabetes	4.2	0.995‒17.5	0.051
Curve shape (down sloping and up sloping; flat profound and flat moderate to severe)	9.3	2.4‒36	0.001
Onset of therapy (≤ a; > 3)	3	0.978‒9.2	0.055

a Reference level.

We confirmed a statistically significant association between tonal audiometric results (down sloping and up sloping; flat profound and flat moderate to severe) and diabetes with recovery (complete and incomplete). Onset of therapy is associated with recovery on a scale of significance of 94.5%.

In Table 4, associative risk factors are compared with extent of recovery (no recovery, partial recovery, and complete recovery).

**Table 4:** Associative risk factors with recovery (no recovery, partial recovery, and complete recovery).

	Recovery			
	No recovery (n = 19)	Partial recovery (n = 25)	Complete recovery (n = 31)	*p*
Gender; n(%)				0.794
Males	10 (53)	21 (62)	19 (61)	
Females	9 (47)	13 (38)	12 (39)	
Age (years) median (Q1‒Q3); min‒max	61 (54‒72; 30‒87)	57 (43‒67; 8‒80)	52 (44‒59; 27‒81)	0.086
Dislipidemia	12 (63)	23 (68)	17 (55)	0.564
Hypertension	11 (58)	16 (47)	17 (55)	0.707
Diabetes	10 (53)	9 (26)	3 (10)	0.004
Curve shape				<0.001
Flat profound	13 (68.4)	9 (26.5)	3 (9.7)	
Down sloping	1 (5.3)	17 (50)	14 (45.2)	
Up sloping	3 (15.8)	4 (11.8)	14 (45.2)	
Flat moderate to severe	2 (10.5)	4 (11.8)	0	
Onset of therapy				0.069
≤ 3 (days)	11 (60)	17 (50)	24 (77)	
> 3 (days)	8 (40)	17 (50)	7 (23)	

Upon study, neither the gender nor the age of the patients was a statistically significant predictive factor for specific recovery expectations (gender: χ^2^ = 0.486; *p* = 0.784) (age: χ^2^ = 5.4; *p* = 0.086). Additionally, neither dyslipidemia (χ^2^ = 1.14; *p* = 0.564) nor hypertension (χ^2^ = 0.693; *p* =  0.707) was a statically significant factor associated with predicting recovery.

There are 2× more diabetic patients in the group no recovery than in the group partial recovery (OR = 3.1; 95% CI 0.95‒10; *p* = 0.061), and 5.3× more than in the group complete recovery (OR = 10.4; 95% CI 2.3‒45; *p* = 0.002) (χ^2^ = 11.2; *p* = 0.004).

Patient categorization according to tonal audiometric curve results statistically differed in terms of recovery (complete, partial, no recovery) (χ^2^ = 32.5; *p* < 0.001). Flat profound curve type contributes the most to the difference, which has 7× more cases of no recovery than complete recovery. Patients with down sloping curve resulted in 10× less cases of no recovery compared with either complete or partial recovery.

Patients with upward sloping curve experienced 3.5× more cases of complete recovery compared with either no or partial recovery.

There is a statistically significant correlation between onset of therapy and recovery on a scale of significance of 93% (χ^2^ = 5.3, *p* = 0.069).

Median onset of therapy for cases with complete recovery is 2 days (q1‒3:1‒3.5; min‒max: 1‒7), for cases of no recovery is 3 days (q1‒q3 1‒7; min‒max: 1‒21), and for cases of partial recovery is 3.1 days (Q1‒Q3: 2‒6; min‒max: 1‒10).

## Discussion

Our research aimed to discover the impact of certain clinical parameters on the likelihood of hearing recovery after SSNH. In addition to this, we aimed to detect potential variables that could predict the disease outcome. We found that neither the gender nor the age of the patients were statistically significant predictive factors for specific recovery expectations.

The cochlea is a peripheral organ receiving blood only through the labyrinthine artery and with weak collateral blood circulation. Thus, the possibility that SSNHL occurs because of an interruption in the blood flow to the cochlea.[13]

Because an interrupted blood flow and temporary ischemia have been suggested as the pathophysiology, several studies reported that patients with cardiovascular risk factors such as dyslipidemia, diabetes and hypertension have an unfavorable prognosis in SSNHL.[14]

According to our data, neither dyslipidemia and hypertension were statically significant factors associated with predicting the resolution.

Diabetes Mellitus was shown to be a statistically significant negative predictive factor for recovery. Our study shows 2× more diabetic patients in the group no recovery than in the group incomplete recovery and 5.3× more than in the group complete recovery.

Metabolic syndrome and diabetes mellitus had a increased risk of having sudden sensorineural hearing loss in other scientific papers.[15], [16], [17], [18], [19] It also correlated with a poor prognosis.[17], [18], [19]

Hyperglycemia initiates an array of functional pathologies that include the disruption of mitochondrial DNA. Affected mitochondria will impair oxidative phosphorylation and ATP production that will preferentially contribute to dysfunction of the stria vascularis of the inner earr. In diabetes, endothelial dysfunction seems to play a role in the initiation of microvascular pathology that includes thickening of the basement membrane, pericyte degeneration, and endothelial cell hyperplasia.[20]

According to our research, tonal audiometric test result is statistically significant predictive factor for hearing loss recovery. Patient categorization according to tonal audiometric curve results statistically differed in terms of recovery (complete, incomplete, no recovery). Flat profound curve shape contributes the most to the difference, which has 7× more cases of no recovery than complete recovery. Complete recovery rate was significantly lower in patients with profound hearing loss in Edizer et al. work.[21]

If the severity of initial hearing loss is high, the recovery rate is low. It is believed that in cases of profound loss, the extent of hair cell injury is so extensive, it does not allow a significant structural and functional recovery.[10]

Our research found that there is a statistically significant correlation between onset of treatment and recovery on a scale of significance of 93%. In addition, cases of incomplete recovery are 3.1× more often found in patients who began therapy after three days compared with those patients who began within 3 days.

This leads us to the conclusion that the sooner therapy is begun, the higher chance a patient has for better recovery.

Other authors also concluded that the recovery was significantly related to the onset of hearing loss. Delayed therapy often leads to permanent irreversible damage of inner ear that cannot lead to hearing recovery.[10], [22], [23]

Limitation of the study is a small sample size. Further studies with a larger number of patients are warranted to contribute to strenth of the study.

## Conclusion

In our study tonal audiometry results, diabetes mellitus and onset of therapy were shown to be a statistically significant negative predictive factors for recovery. Results of pure tone audiometry suggest a flat profound curve is statistically associated with the no recovery rates.

## Funding

This research did not recieve any specific grant from funding agencies in the public, commercial, or not-for-profit sectors.

## Conflicts of interest

The authors declare no have conflicts of interest.
